# Supplementary material for: Discoidin-domain receptor coordinates cell-matrix adhesion and collective polarity in migratory cardiopharyngeal progenitors
Source: Nat Commun. 2019 Jan 4;10:57. doi: 10.1038/s41467-018-07976-3 (PMC6320373; doi:10.1038/s41467-018-07976-3)
Supplement: Supplementary file 1 — Supplementary Information [file 41467_2018_7976_MOESM1_ESM.pdf]

Supplementary Figure 1

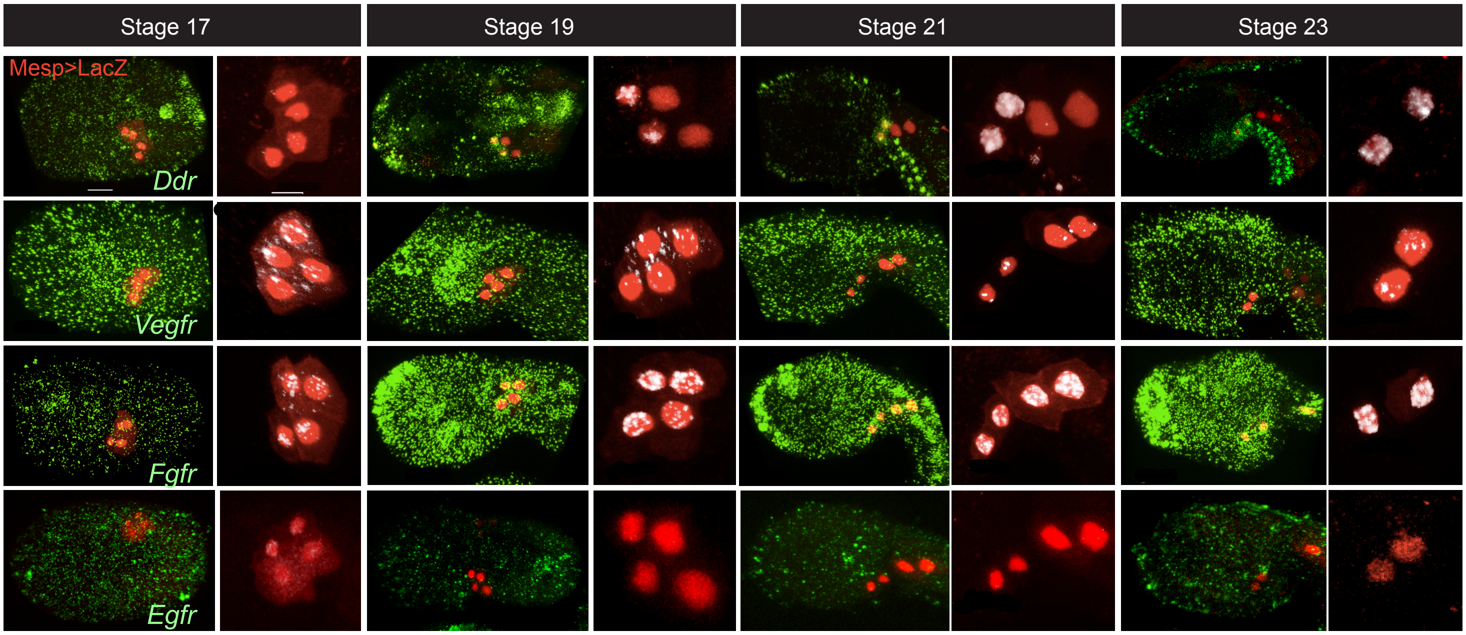

Supplemental Figure 1. RTK expression during TVC migration. Fluorescent *in situ* hybridization for *Ddr, Vegfr, Fgfr,* and *Egfr* at indicated developmental stages. B7.5 lineage is marked with *Mesp>LacZ* and stained for beta-galactosidase (red in the micrographs). Close ups show colocalization of transcripts with B7.5 lineage nuclei. Scale bar whole embryo = 20um, B7.5 lineage closeup = 10um.

Supplementary Figure 2

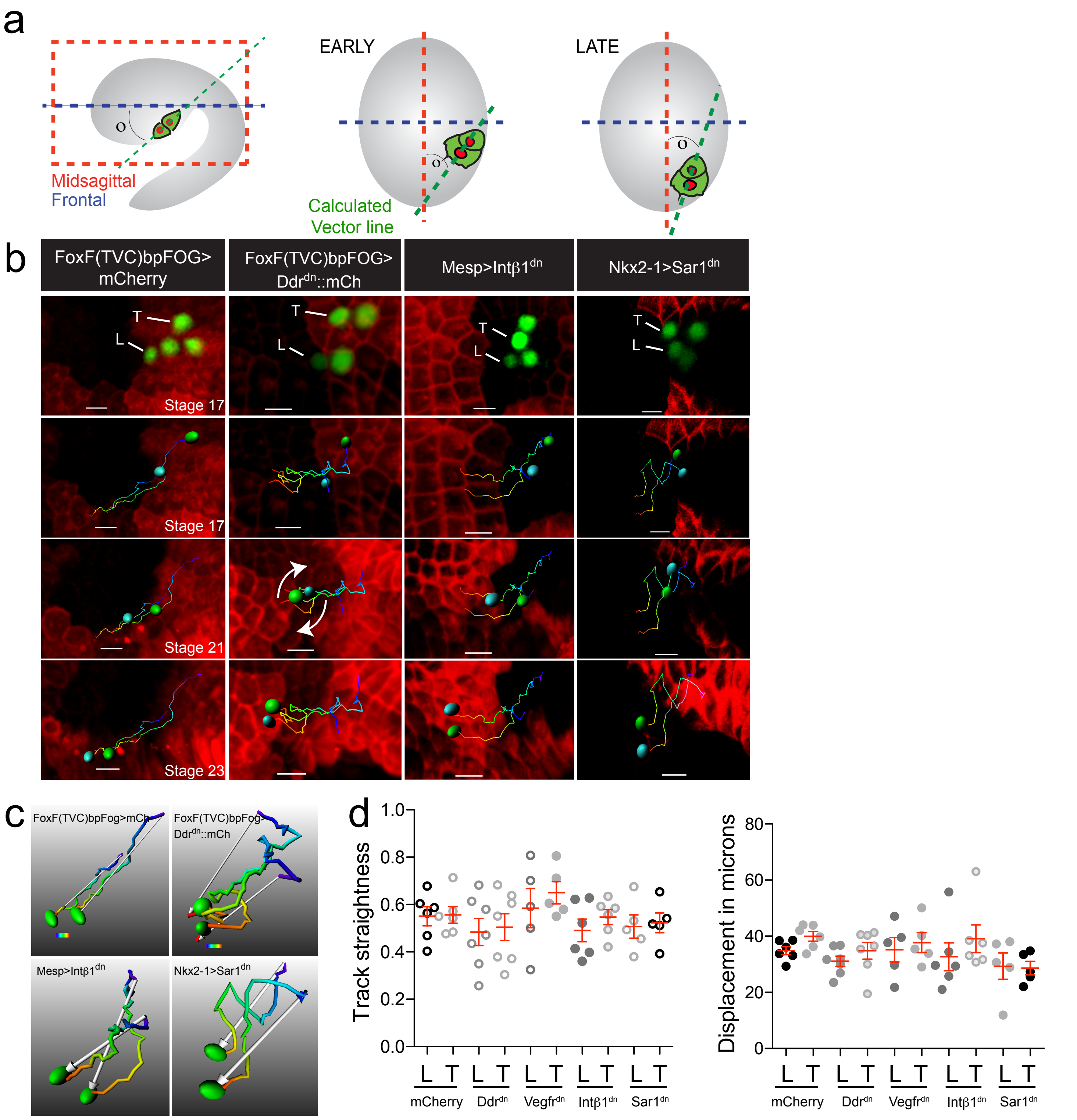

Supplemental Figure 2. Quantitative analysis of TVC migration. a. Schematic of calculating TVC position relative to frontal (blue) or sagittal (red) planes. The vector line (green) is calculated using the axes of the TVC nuclei. **b.** Positional tracking of TVC migration under perturbations of ECM adhesion. Graphs show average positions of TVCs relative to orthogonal planes and standard deviation at each time point. Scale bar = 10um **c.** Rendered images of total displacement of the TVC under adhesion perturbation. White arrows point to the final position of TVCs prior to the first asymmetric division. TVC tracks are time code blue for early time points and red for late time points. **d.** Average track straightness and total displacement of TVCs. Error bars show S.E.M.

Supplementary Figure 3

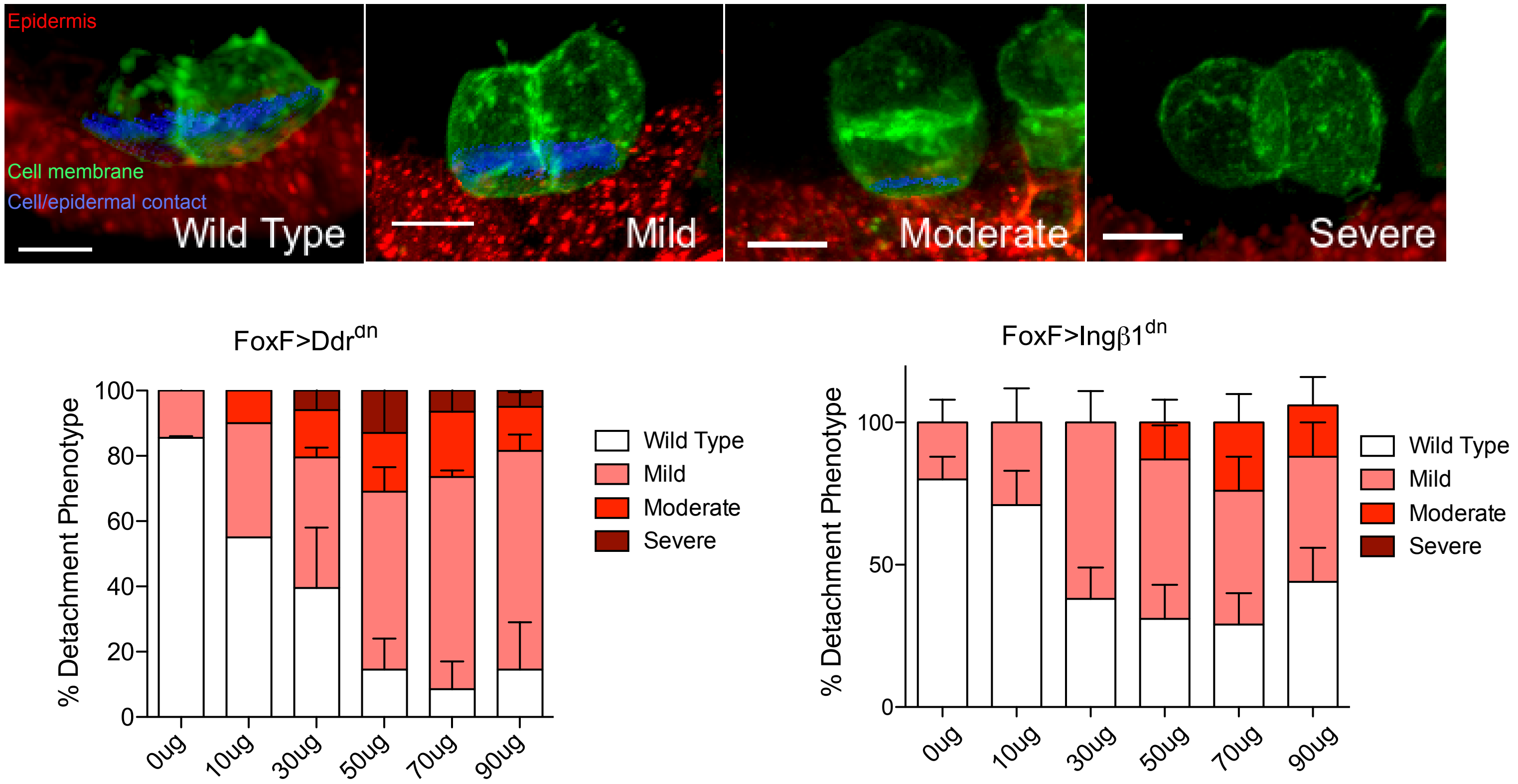

Supplemental Figure 3. Dose response to adhesion perturbation. Micrographs show the range of phenotypes associated with adhesion defects ranging from wild type, where leader and trailer maintain epidermal contact, mild, where epidermal contact is reduced, moderate, where either the leader or the trailer loses contact with the epidermis, and severe, where both leader and trailer lose contact with the depidermis. Cell membrane is marked with Mesp>hCD4::GFP, epidermis is marked with EfnB>CD4::RFP. Epidermal contacts are shown in blue. Graphs show increasing penetrance of detachment phenotype, as a function of increasing dominant negative loading. Error bars show standard error of proportion. Scale bar = 10um.

Supplementary Figure 4

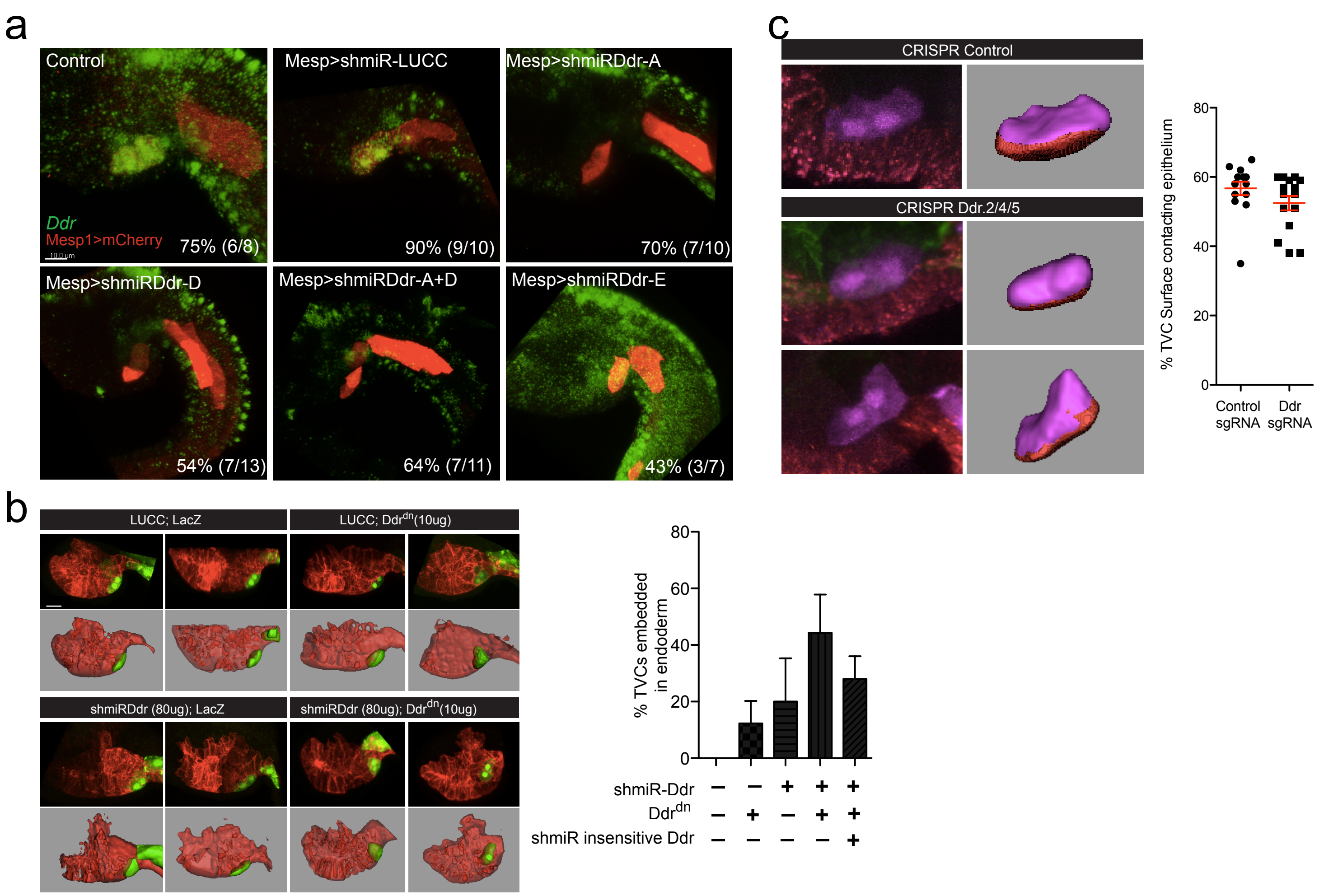

Supplementary Figure 4. Ddr loss-of-function phyenotypes. **a.** Short hairpin mircroRNA (shmiR) Knockdown of Ddr transcipts. B7.5 lineage is marked with *Mesp>mCherry*. Scale bar = 10um. **b.** Sensitized shmiR test. Two representative embryos are shown for each condition. Top rows show raw data, bottom rows show rendered images. Graph shows increased contact of TVCs with the endoderm as TVCs detach from the epidermis and increased percentage of TVCs embedded in the endoderm. Scale bar = 20um. **c.** Mutagenesis of the *Ddr* locus using CRISPR. *EBF* CRISPR is used as control. Graph shows average percent of the TVC pair surface in contact with the epidermis. S.E.M. is shown

Supplementary Figure 5

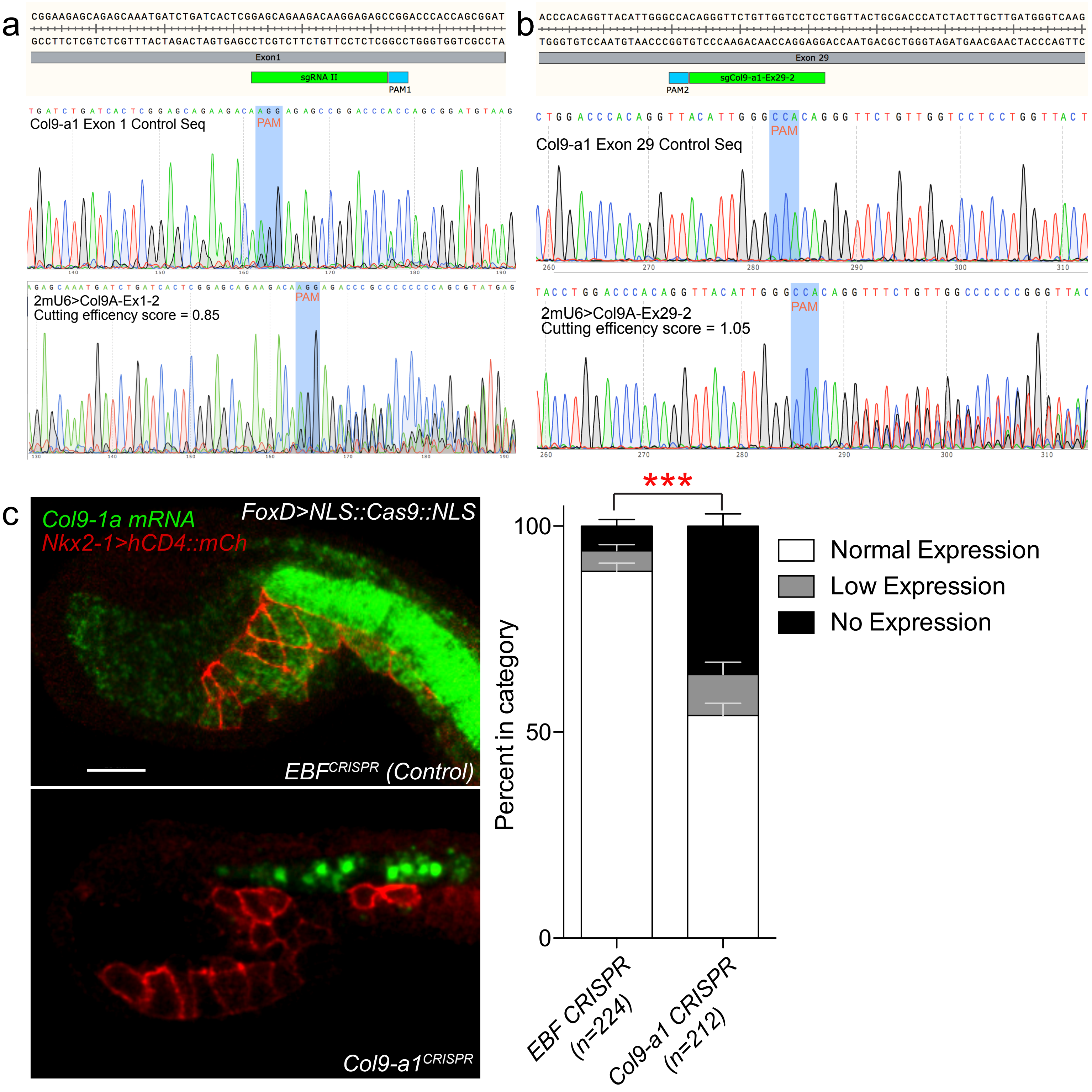

Supplementary Figure 5. Col9-a1 CRISPR guide RNA cutting efficency
Peak shift analysis as estimate of cutting efficency (**a**,**b**). Upper sequence panel shows targeting guide RNA s equence. Wild type sequence and peak shifts produced by CRISPR targeting of the locus are shown. Efficiency scores are calculated by adding the quality scores of each base position and normalizing by the uncut sequence. **c** *Col9-a1* in situ in control embryos with CRISPR targeting the *EBF* locus and CRISPR targeting the *Col9-a1* locus. Note mosaic expression of the Nkx2-1>hCD4::mCherry reporter in the endoderm. Standard Error of proportion is shown and significance established using the Chi-square test. ***=p<0.0005. Scale bar = 20.

Supplementary Figure 6

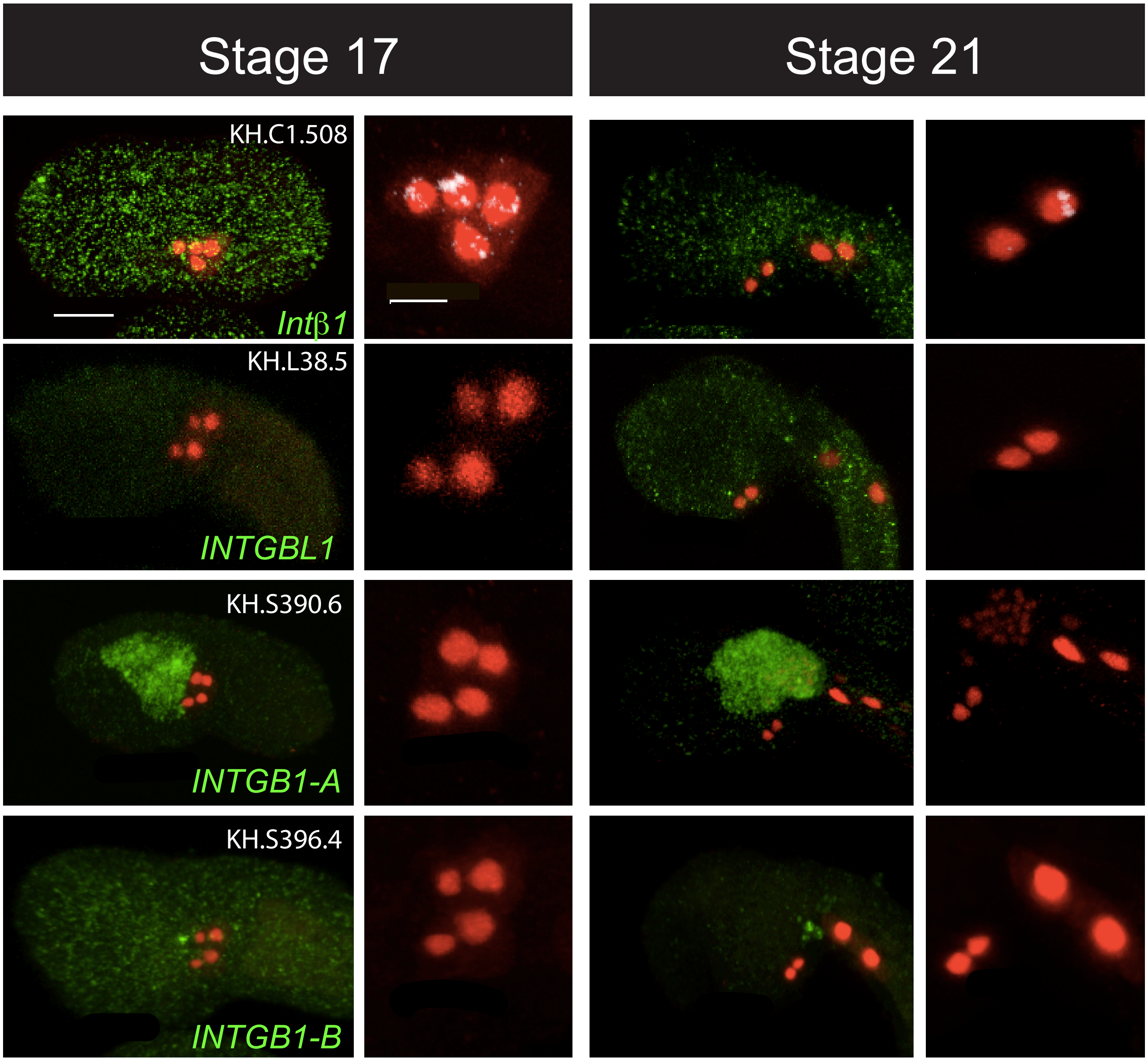

Supplementary Figure 6. Integrin expression in the B7.5 lineage. In situ hybridization of RNA probes against indicated transcripts. B7.6 lineage is marked with *Mesp>LacZ* and stained for β-gal. Scale bar of whole embryo = 30um, scale bar for closeups = 15um.

Supplementary Figure 7

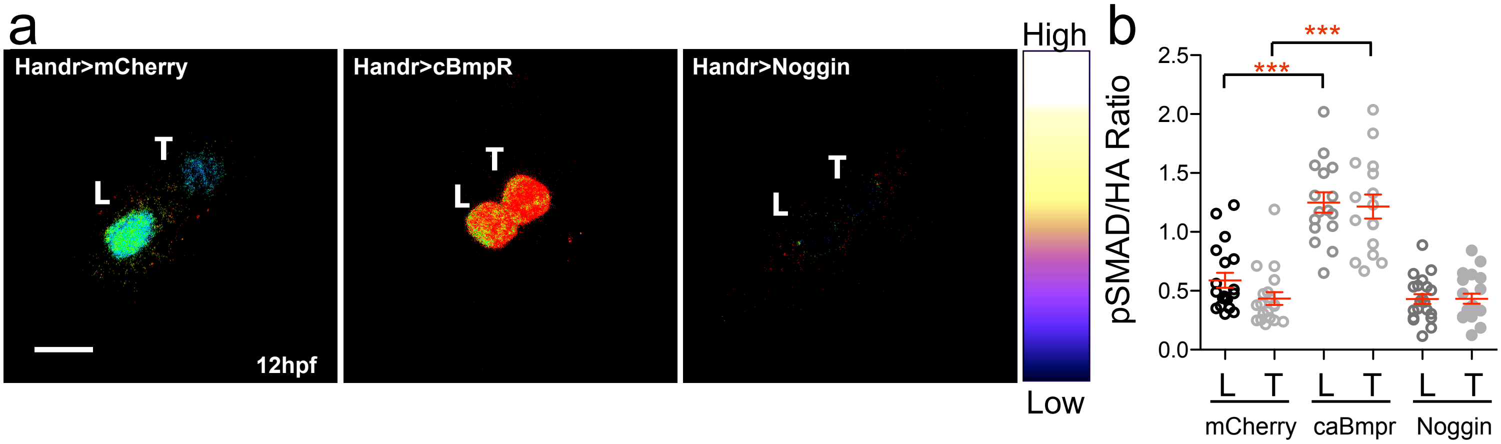

Supplementary Figure 7. Asymmetric induction of BMP-pSmad activity in migrating TVCs. a. Ratios of detected pSmad staining to the total HA levels detected. Levels can be manipulated through addition of a consitituitivley active BMP receptor (Handr>caBmpR) or by expression of the BMP antagonist Nogging (Handr>Noggin). Scale bar = 5um **b.** Calculated ratios of pSmad/HA under conditions altering BMP-pSmad activity levels. Statistical analysis using a two-way ANOVA. S.E.M. is shown.

Supplementary Figure 8

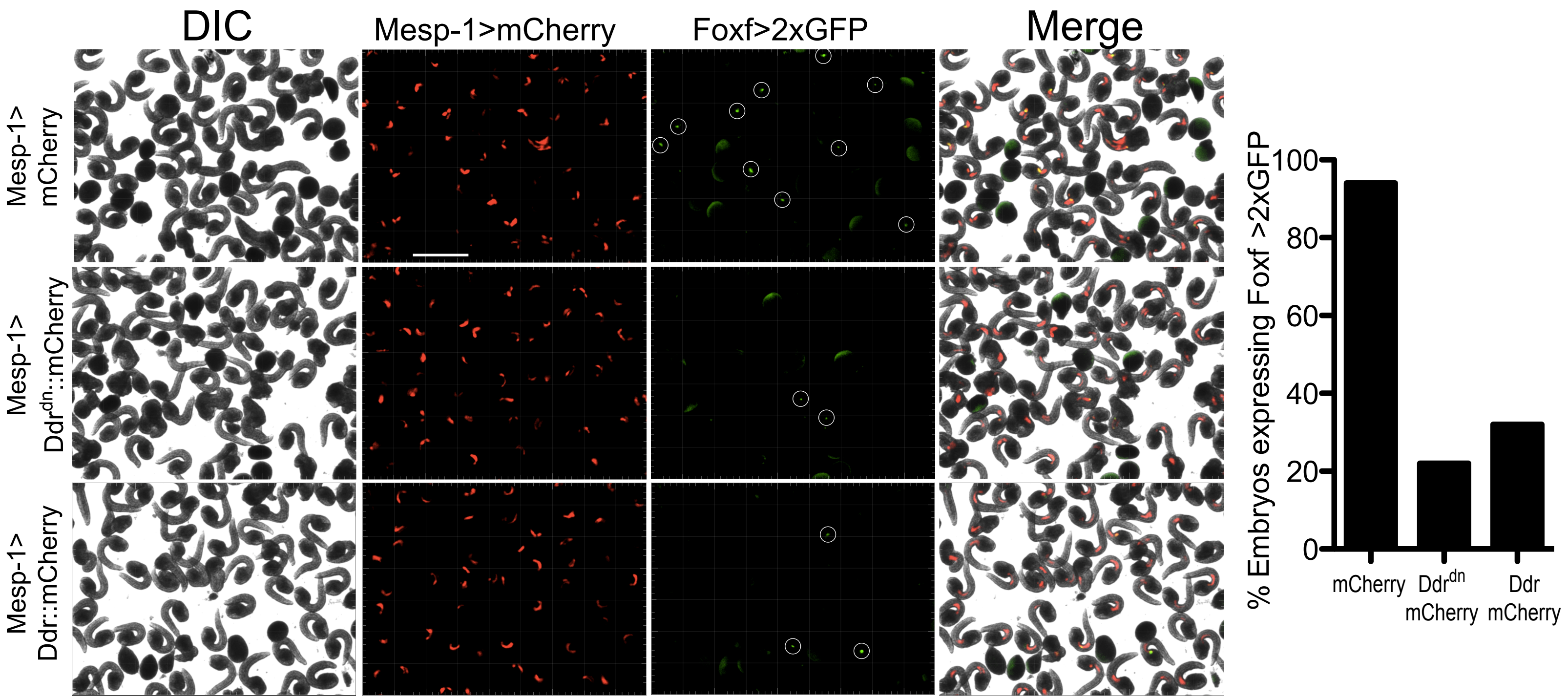

Supplementary Figure 8. Early expression of Ddr blocks TVC fate induction. Embryos electroporated with Mesp-driven mCherry, a dominant negative Ddr (Ddr^{dn}) or full-length Ddr are assayed for expression of Foxf-driven GFP. DIC channel shows embryonic developmental stage. Foxf>2xGFP expressing TVCs are circled in the green channel. Scale bar = 100um.

Primers for full-lenfth cloning

Gene	Forward Primer	Reverse Primer
Vegfr-F	acttgtattGCGGCCGCAACCATGAAACTAACTGTCCAACTTGTTCTGATC	TCACCATGGTGCTAGCATTGAGATCCTGAATCCTGGAAGC
Egfr-F	acttgtattGCGGCCGCAACCATGAGGCATTGCTATCCAATAGTTG	TCACCATGGTGCTAGCCGTTTGTGGGTGGCCAC
Fgfr-F	acttgtattGCGGCCGCAACCATGATACAACTACAAAATACGTTTATTTTTGTCG	TGCTCACCATACTAGTAAAAACAATACCAAGAATAATATGAAAACCCACC
Ddr-F	acttgtattGCGGCCGCAACCATGAAGTCGAAGTCATTACCACCGAGT	TGCTCACCATACTAGTCGCTCTTGGGGTGGGAATTGG
Col9-a1-F	ATGCGGCCGCAACCATGTTCATGAACAAGAGGCGA	ATACTAGTAAACTGAATATCATTCATCTC
Ci-shmiR-insensitive Ddr	CGAGGTAGAGTTTGACAATAACGTATTACCACCGAGCGAT	TATTGTCAAACTCTACCTCGCTAACCAGAAGATATTCGTCCCC

Primers for generatiang dominant negatives

Gene	Primer
dnDDR-R	TGCTCACCATACTAGTCTTCTCACGAGGAAACTCGGG
dnVEGFR-R	TGCTCACCATACTAGTTCGATCTCGAGGAAACTCCCATTTC
dnFGFR-R	CCACTACAACAAGCCCTTGATTCTTAAGG
dnEGFR-R	TGCTCACCATACTAGTTTCGCTTTCTTTAACAATTCTAAGTTGAGC
dnIntβ1-F	AACACACAAGCGGCCGCAACCATGTGTGATATACATAAAGATTGCATTCAATGCAA
dnIntβ1-R	CGCTCAGCTGGAATTCCCATATAAGCAGAATAGCAAGCCCA

In Situ Primer list

Gene	Forward Primer	Reverse Primer
Col9-a1	ATGCGGCCGCAACCATGTTCATGAACAAGAGGCGA	ATACTAGTAAACTGAATATCATTCATCTC
Vegfr	ATGAAACTAACTGTCCAACTTGTTCTGATC	ATTGAGATCCTGAATCCTGGAAGC
Fgfr	ATGATACAACTACAAAATACGTTTATTTTTGTCG	TAGTGTTAATACCAGCTCTCTTCAATGTAATCAC
Egfr	ATGAGGCATTGCTATCCAATAGTTG	AGTTATTGTGGATGTGGGGTTCATTTG
Intβ1	acttgtattGCGGCCGCAACCATGAAAATGAATTATATTACAGTGTTATATTGGTGTG	ACTGGTAGTTACAGCAAAGATTGGTTG
Vegf	GCGGCCGCaaccATGCACCAGGATTTAAGAGTCAAATCTC	GAATTCACTAGTTTTTCCCCTGGCGAAATGTCC

Ci_shmiR Primers

Gene	Forward Primer	Reverse Primer
LUCC-C	agatGGGATTTCctTtGATGTAttTGTAATTaGtAACATGTACATCGACTGAAATCttttt	aattaaaaaGATTTCAGTCGATGTACATGTTaCtAATTACAaaTACATCaAagGAAATCCC
Ddr-miR-A	gtttGGATAGCAccGtTGGttAAcTGTAATTaGtAACATTTGTCCAGCAATGCTATCC	ttagGGATAGCATTGCTGGACAAATGTTaCtAATTACAgTTaaCCAaCggTGCTATCc
Ddr-miR-B	gtttGAAATGCGctTTtATTGTAcTGTAATTaGtAACAtTACAATGAACTCGCATTCT	ttagAGAATGCGAGTTCATTGTAaTGTTaCtAATTACAgTACAATaAAagCGCATTTC
Ddr-miR-C	gtttGGTTGGTAgaGTATGAtATcTGTAATTaGtAACATATGTCATACGATACCAATC	ttagGATTGGTATCGTATGACATATGTTaCtAATTACAgATaTCATACtcTACCAACC
Ddr-miR-D	gtttGCAAGTCGtgTTCGATAAttTGTAATTaGtAACATGTTATCGAATTCGACTTCG	ttagCGAAGTCGAATTCGATAACATGTTaCtAATTACAaaTTATCGAAcaCGACTTGC
Ddr-miR-E	gtttGGTGGTCCtcATtGAAATttTGTAATTaGtAACATGATTTCGATCTGGACCAAC	ttagGTTGGTCCAGATCGAAATCATGTTaCtAATTACAaaATTTCaATgaGGACCACC
Ddr-miR-F	gtttGGCGGTACgtTATCAAtTAcTGTAATTaGtAACATTAGTTGATATGGTACCGTC	ttagGACGGTACCATATCAACTAATGTTaCtAATTACAgTAaTTGATAacGTACCGCC

Col9-a1 sgRNAs

Target	Forward Oligo	Regerse Oligo
Col9-a1-Ex1	GAGCAGAAGACAAGGAGAGCGTTTAAGAGCTATGCTGGAAACAG	GCTCTCCTTGTCTTCTGCTCatctataccatcggatgccttc
Col9-a1-Ex29	GGAGGACCAACAGAACCCTGGTTTAAGAGCTATGCTGGAAACAG	CAGGGTTCTGTTGGTCCTCCatctataccatcggatgccttc

Supplementary Table 1. Primers used for generation of full length, dominant negatives, in situ probes, shmiRs, and guide RNAs.
