# Supplementary material for: Discoidin-domain receptor coordinates cell-matrix adhesion and collective polarity in migratory cardiopharyngeal progenitors
Source: Nat Commun. 2019 Jan 4;10:57. doi: 10.1038/s41467-018-07976-3 (PMC6320373; doi:10.1038/s41467-018-07976-3)
Supplement: Supplementary file 3 — Description of Additional Supplementary Files [file 41467_2018_7976_MOESM3_ESM.pdf]

Supplementary Movie Legends

Supplementary Movie 1. Tracking of TVC migration in control conditions. Epidermis is marked with *EfnB1>hCD4::mCh*, TVC nuclei are marked with *Mesp>H2B::GFP*. Temporal step = 3.4 minutes.

Supplementary Movie 2. Tracking of TVC migration under Ddr^{dn} conditions. Epidermis is marked with *EfnB1>hCD4::mCh*, TVC nuclei are marked with *Mesp>H2B::GFP*. Temporal step = 3.4 minutes

Supplementary Movie 3. Tracking of TVC migration under Vegfr^{dn} conditions. Epidermis is marked with *EfnB1>hCD4::mCh*, TVC nuclei are marked with *Mesp>H2B::GFP*. Temporal step = 3.4 minutes

Supplementary Movie 4. Tracking of TVC migration under Fgfr^{dn} conditions. Epidermis is marked with *EfnB1>hCD4::mCh*, TVC nuclei are marked with *Mesp>H2B::GFP*. Temporal step = 3.4 minutes

Supplementary Movie 5. Tracking of TVC migration under Egfr^{dn} conditions. Epidermis is marked with *EfnB1>hCD4::mCh*, TVC nuclei are marked with *Mesp>H2B::GFP*. Temporal step = 3.4 minutes
