# Supplementary material for: Discoidin-domain receptor coordinates cell-matrix adhesion and collective polarity in migratory cardiopharyngeal progenitors
Source: Nat Commun. 2019 Jan 4;10:57. doi: 10.1038/s41467-018-07976-3 (PMC6320373; doi:10.1038/s41467-018-07976-3)
Supplement: Supplementary file 9 — Reporting Summary [file 41467_2018_7976_MOESM9_ESM.pdf]

nature research

Corresponding author(s): Lionel Christiaen (lc121@nyu.edu)

Reporting Summary

Nature Research wishes to improve the reproducibility of the work that we publish. This form provides structure for consistency and transparency in reporting. For further information on Nature Research policies, see Authors & Referees and the Editorial Policy Checklist.

Statistical parameters

When statistical analyses are reported, confirm that the following items are present in the relevant location (e.g. figure legend, table legend, main text, or Methods section).

n/a	Confirmed	
☐	☒	The exact sample size (n) for each experimental group/condition, given as a discrete number and unit of measurement
☐	☒	An indication of whether measurements were taken from distinct samples or whether the same sample was measured repeatedly
☐	☒	The statistical test(s) used AND whether they are one- or two-sided<br>*Only common tests should be described solely by name; describe more complex techniques in the Methods section.*
☐	☒	A description of all covariates tested
☒	☐	A description of any assumptions or corrections, such as tests of normality and adjustment for multiple comparisons
☐	☒	A full description of the statistics including central tendency (e.g. means) or other basic estimates (e.g. regression coefficient) AND variation (e.g. standard deviation) or associated estimates of uncertainty (e.g. confidence intervals)
☒	☐	For null hypothesis testing, the test statistic (e.g. F, t, r) with confidence intervals, effect sizes, degrees of freedom and P value noted<br>*Give P values as exact values whenever suitable.*
☒	☐	For Bayesian analysis, information on the choice of priors and Markov chain Monte Carlo settings
☒	☐	For hierarchical and complex designs, identification of the appropriate level for tests and full reporting of outcomes
☒	☐	Estimates of effect sizes (e.g. Cohen's d, Pearson's r), indicating how they were calculated
☐	☒	Clearly defined error bars<br>*State explicitly what error bars represent (e.g. SD, SE, CI)*

Our web collection on statistics for biologists may be useful.

Software and code

Policy information about availability of computer code

Data collection	Leica SP8 X WLL Confocal microscope was used for all data collection.
Data analysis	Bitplane Imaris version 9.2.1 was used for colocalization, spot tracking, and surface area calculation.

For manuscripts utilizing custom algorithms or software that are central to the research but not yet described in published literature, software must be made available to editors/reviewers upon request. We strongly encourage code deposition in a community repository (e.g. GitHub). See the Nature Research guidelines for submitting code & software for further information.

Data

Policy information about availability of data

All manuscripts must include a data availability statement. This statement should provide the following information, where applicable:

- Accession codes, unique identifiers, or web links for publicly available datasets
- A list of figures that have associated raw data
- A description of any restrictions on data availability

The full data supporting this article are available from the corresponding author upon reasonable request.

Field-specific reporting

Please select the best fit for your research. If you are not sure, read the appropriate sections before making your selection.

☒ Life sciences ☐ Behavioural & social sciences ☐ Ecological, evolutionary & environmental sciences

For a reference copy of the document with all sections, see nature.com/authors/policies/ReportingSummary-flat.pdf

Life sciences study design

All studies must disclose on these points even when the disclosure is negative.

Sample size	Although no sample size calculation was done prior to experiments we aimed for sample sizes n=30-50 whenever possible. We are able to detect statistically different means and provide biological replicates.
Data exclusions	No data were excluded.
Replication	At least wo biological replicates were used for each experiment.
Randomization	Randomization was performed at the time of the electroporation, which randomly distributes fertilized eggs into experimental groups.
Blinding	Blinding was not possible as a single person performed the experiments.

Reporting for specific materials, systems and methods

Materials & experimental systems

n/a	Involved in the study
☒	☐ Unique biological materials
☐	☒ Antibodies
☒	☐ Eukaryotic cell lines
☒	☐ Palaeontology
☐	☒ Animals and other organisms
☒	☐ Human research participants

Methods

n/a	Involved in the study
☒	☐ ChIP-seq
☒	☐ Flow cytometry
☒	☐ MRI-based neuroimaging

Antibodies

Antibodies used	Rabbit Anti-HA (Abcam ab9110), mouse anti-pTyr (Millipore 4G10 Platinum anti-Phosophotyrosine, Sigma-Aldrich, Cat # 05-321x), rat anti-HA antibody (Roche 11-867-423-001), Polyclonal Rabbit anti-phospho-SMAD5 (Cell Signaling, 9516S, 1:250) , mouse anti-beta-galactosidase (Promega 23781 1:500)
Validation	mouse anti-beta-galactosidase (Promega 23781 1:500) used in Gline et al., 2015,<br>Polyclonal Rabbit anti-phospho-SMAD5 (Cell Signaling, 9516S, 1:250) validated in Supplemental Figure 7.<br>rat anti-HA antibody (Roche 11-867-423-001), cited in Jung et al., 2015<br>mouse anti-pTyr (Millipore 4G10 Platinum anti-Phosophotyrosine, Sigma-Aldrich, Cat # 05-321x), validated in our Duolink PLA experiments<br>Rabbit Anti-HA (Abcam ab9110), cited in Seitz et al., 2014.

Animals and other organisms

Policy information about studies involving animals; ARRIVE guidelines recommended for reporting animal research

Laboratory animals	The study did not involve laboratory animals
Wild animals	This study did not involve wild animals.
Field-collected samples	Ciona robusta specimens were ordered from M-Rep, San Diego (http://m-rep.com/index.htm).
